# Protein-Stabilized Emulsion Gels with Improved Emulsifying and Gelling Properties for the Delivery of Bioactive Ingredients: A Review

**DOI:** 10.3390/foods12142703

**Published:** 2023-07-14

**Authors:** Yuan Xu, Liping Sun, Yongliang Zhuang, Ying Gu, Guiguang Cheng, Xuejing Fan, Yangyue Ding, Haotian Liu

**Affiliations:** 1 Faculty of Food Science and Engineering, Kunming University of Science and Technology, Kunming 650500, China; 20212214082@stu.kust.edu.cn (Y.X.); lpsun@kmust.edu.cn (L.S.); ylzhuang@kmust.edu.cn (Y.Z.); guying@kust.edu.cn (Y.G.); ggcheng@kmust.edu.cn (G.C.); fanxuejing@kust.edu.cn (X.F.); 2 College of Food Science, Northeast Agricultural University, Harbin 150030, China

**Keywords:** protein, emulsion gel, emulsifying and gelling properties, modification, bioactive ingredients delivery

## Abstract

In today’s food industry, the potential of bioactive compounds in preventing many chronic diseases has garnered significant attention. Many delivery systems have been developed to encapsulate these unstable bioactive compounds. Emulsion gels, as colloidal soft-solid materials, with their unique three-dimensional network structure and strong mechanical properties, are believed to provide excellent protection for bioactive substances. In the context of constructing carriers for bioactive materials, proteins are frequently employed as emulsifiers or gelling agents in emulsions or protein gels. However, in emulsion gels, when protein is used as an emulsifier to stabilize the oil/water interface, the gelling properties of proteins can also have a great influence on the functionality of the emulsion gels. Therefore, this paper aims to focus on the role of proteins’ emulsifying and gelling properties in emulsion gels, providing a comprehensive review of the formation and modification of protein-based emulsion gels to build high-quality emulsion gel systems, thereby improving the stability and bioavailability of embedded bioactive substances.

## 1. Introduction

With the progress of society, the prevalence of obesity and various chronic diseases caused by unhealthy lifestyles, such as insufficient exercise, inadequate rest, and excessive intake of high-calorie foods, is on the rise. As a result, bioactive compounds (such as polyphenols, carotenoids, and polyunsaturated fatty acids) are becoming increasingly popular for their potential in preventing or even treating such chronic diseases [1]. However, the chemical instability of these bioactive compounds can lead to their loss and degradation when exposed to environmental factors such as light, heat, and oxygen, thereby reducing their bioavailability [2]. Consequently, the development of delivery systems capable of encapsulating bioactive compounds, such as emulsions [3], hydrogels [4], and emulsion gels [5], has attracted considerable attention in the field of functional foods and biomedical preparations.

Among these delivery systems, emulsion gels, which mitigate oil movement and oxygen diffusion, are considered to offer superior stability for encapsulating bioactive substances. Additionally, the presence of an oil phase allows for the dissolution of lipophilic bioactive compounds [6]. Therefore, emulsion gels can serve as effective carriers for unstable bioactive ingredients. Due to their solid nature and structured systems, emulsion gels form a three-dimensional network structure that immobilizes dispersed phases within the gel structure [7]. This unique gel-like structure, coupled with strong mechanical properties, can provide robust protection for bioactive substances in adverse environmental conditions. Several studies have reported that emulsion gels can improve the stability of β-carotene [8], α-tocopherol [9], and curcumin [10], while also playing an important role in controlling the release of volatile substances such as propanol, diacetyl, pentanone, hexanal, and heptanone [11].

Previous studies have highlighted the significance of emulsifiers, gelling agents, oil phases, and preparation conditions (e.g., homogenization methods and ambient pressure) in shaping the structures and functionalities of emulsion gel systems [3]. Emulsifiers, in particular, serve as key components in the formation and stability of emulsion gels. Numerous studies have explored the development of naturally degradable, food-grade particle emulsifiers, with proteins and polysaccharides being widely utilized due to their inherent emulsifying and/or gelling properties [2,12]. Currently, the most commonly used examples in the food industry are animal and plant proteins, and polysaccharides such as gum arabic, pectin, and galactomannan [12,13]. Polysaccharides (mostly having hydrophilic nature) are a large group of high molecular weight biopolymers that are utilized as thickeners, gels, emulsifiers, and stabilizers in food, pharmaceutical, and cosmetic products [14]. Proteins, as naturally amphiphilic polymers, are prone to adsorption at oil/water interfaces, leading to the formation of an interfacial film [15]. Furthermore, proteins also have various types of functional groups on their surface, which can bind to different types of molecules through hydrogen bonding, hydrophobic interactions, and electrostatic interactions [16,17]. Therefore, proteins are very versatile when used as emulsifiers to stabilize emulsions or as gelling agents to form protein gels [17]. However, as a complex colloid, emulsion gels can exist simultaneously as both emulsions and gels. In emulsion-filled gels, a continuous phase (such as protein-based gel) forms a continuous gel matrix in which emulsion droplets are embedded, akin to a rubber material filled with droplets. Proteins not only function as emulsifiers to stabilize the emulsion but also act as gelling agents within the gel. The emulsion droplets in emulsion gels aggregate to create a network structure, with approximately all available emulsifiers located at the oil/water interface, and their properties are influenced by the network properties of the aggregated emulsion droplets [18]. 

Therefore, the emulsifying and gelling properties of emulsifiers are particularly important when designing emulsion gels. Current research extensively explores proteins with improved emulsifying and gelling properties for the preparation of high-quality emulsion gels [19,20,21,22,23]. Previous studies have highlighted the significant impact of differences in emulsifying and gelling properties on the structure and performance of emulsion gels. For example, Lin, Kelly, Maidannyk, and Miao (2021) reported that the emulsion exhibited higher viscosity, smaller size, and more uniform droplet distribution when whey protein isolates with higher emulsifying properties were employed [24]. Similarly, in the development of pea protein as an emulsifier for emulsion gels, the emulsifying properties of pea proteins were found to stabilize oil droplets, while their gelling properties during heating aided in forming emulsion gels [25]. This finding was also described by Lu et al. (2020), who prepared emulsion gels using natural whey protein as an emulsifier and heat-denatured whey protein as a gelling agent, resulting in emulsion gels with increased mechanical properties as the content of heat-denatured whey protein was increased [5]. Incorporating bioactive ingredients into these enhanced emulsion gels enhanced their stability and bioavailability [5]. Additionally, the gel structure in the continuous phase enables emulsion gels to be used as multiphase carriers to deliver hydrophilic and lipophilic substances [26]. Therefore, when protein is used as an emulsifier to stabilize the oil/water interface, the structure of the protein gel also has a substantial impact on the functionality of emulsion gels. Thus, it is very crucial to discuss the two important functional properties of emulsification and gelation in proteins when considering their application as emulsifying and/or gelling agents in emulsion gel systems.

This paper mainly reviews the potential of proteins from different sources as emulsifying and/or gelling agents, and provides a comprehensive overview of the formation and modification of protein-based emulsion gels. Special emphasis is placed on the role of protein emulsifying and gelling properties in emulsion gels, along with a detailed discussion of strategies to improve the structure and stability of emulsion gels to build high-quality emulsion gel systems that enhance the bioavailability of embedded bioactive substances.

## 2. Proteins Used as Emulsifiers or Gelling Agents

### 2.1. Animal Proteins

For protein-based food products, animal proteins remain the most widely used because of their excellent stabilizing capacity [27]. Animal proteins often have superior gelling, emulsifying, and foaming properties, and the texture and sensory characteristics of food products are considered superior to those of plant proteins because of these functional properties [28]. Additionally, animal proteins are often considered to have a higher nutritional value than plant proteins because of their amino acid composition and their ability to transport calcium, iron, and other important nutrients [29]. Currently, animal proteins have also been widely investigated as an emulsifier/gelling agent to prepare emulsions or emulsion gels for the encapsulation of bioactive substances [30,31,32]. 

The main sources of animal proteins are dairy products, meat products, eggs, and seafood. Among these, dairy proteins (such as casein and whey protein) are the most commonly used animal proteins due to their availability and can be used as emulsifiers in various products (e.g., ice cream, cheese, and butter) [33]. Approximately 80% of the total protein in milk is casein, which includes α_s1_, α_s2_, β, and κ-casein. These four proteins are all amphiphilic macromolecules (Table 1) [28]. Caseins are highly flexible and unstructured proteins with open and flexible conformations. Their rheomorphic characteristic facilitates absorption into the oil/water interface [34]. Caseins possess several physicochemical properties that make them effective in delivering unstable bioactive components. These properties include the ability to bind ions and small molecules, excellent interfacial stability, exceptional emulsification and self-assembly properties, and excellent water-binding ability [35,36]. Therefore, caseins have great potential for stabilizing emulsions and forming gels. 

Whey protein, another component of milk protein, is widely used in food due to its unique functional properties and high nutritional value. The differences between whey protein and casein lie mainly in their structure and flexibility. Casein has a flexible random coil structure, while whey protein adopts a typical globular protein structure. Globular proteins are spherical structures formed by the dense accumulation of secondary structures, and they are naturally folded into a tertiary structure [50]. In globular proteins, hydrophobic sites are located in the interior, while hydrophilic sites are exposed to water [28,50]. Some reports suggest that once adsorbed at the interface, globular proteins undergo instinctive structural expansion, lateral attractive interaction (between adsorbed proteins), denaturation, and aggregation [50,51]. These abilities are closely related to their emulsifying properties [52]. Whey protein consists mainly of the following four proteins: β-lactoglobulin (β-LG), α-lactalbumin (α-LA), bovine serum albumin (BSA), and lactoferrin (LF) [38]. The structure and functional properties of whey protein mainly depend on its degree of denaturation and changes in the tertiary structure [28,38]. It is worth mentioning that cold-setting gel is commonly used in the induction of whey protein gel to expand its application in food [53,54]. Compared with a heat-setting gel (which maintains a high temperature throughout the process), a cold-setting gel is less prone to induce microphase separation, and the whey protein gel prepared by this method is considered to be stronger and more transparent [50]. This point has also been reported in the previous studies [55,56].

### 2.2. Plant Proteins

With the increasing world population and growing awareness of healthy diets, plant protein is considered a sustainable and promising alternative to animal protein. The trend in food development shows that plant proteins extracted from beans and grains have begun partially or completely replace animal proteins [57]. However, plant protein is not as stable as animal protein, particularly in terms of emulsifying and foaming properties [27]. For example, when pea protein partially replaced whey protein isolate, the stability of foaming and emulsification decreased [58]. More and more studies are focusing on modifying the structures of plant proteins to extend their roles in emulsions, especially in emulsion gels [59,60,61]. 

Plant proteins, such as those derived from legumes (e.g., peas, lentils, chickpeas, and lupines); cereals (e.g., maize, sorghum, wheat, and rice); seeds (e.g., rapeseed, flaxseed, and chia) (Table 1). Storage proteins mainly include albumins, globulins, prolamins, and glutelins [62]. Among the commonly used plant proteins, soybeans and seeds consist mainly of complex globular proteins (including 7S and 11S globulins) and albumin [63]. Take the globular protein in soy protein isolate as an example. Soy protein isolate is the most commonly used legume in an emulsion gel formulation, containing over 90% protein (dry basis) [64]. It mainly comprises β-conglycinin (7S) and glycine globular protein (11S). The 7S globulin (β-conglycinin) is a trimeric glycoprotein composed of the following three subunits: α (67 kDa), α’ (71 kDa), and β (50 kDa) [65]. In 7S globulin, the trimerization of subunits is primarily influenced by hydrophobic interactions, followed by hydrogen bonding and a salt bridge. These interactions are also susceptible to environmental factors such as pH and ionic strength [66]. Glycinin (300–380 kDa), the 11S globulin, is a hexamer composed of five subunits. Each subunit consists of an acidic polypeptide and a basic polypeptide linked together by disulfide bonds [28,65]. The acidic or basic polypeptide within a single hexagon is believed to be formed by hydrogen and/or electrostatic bonding, while the overall structure of 11S is mainly maintained by hydrophobic interactions [66]. 

Some studies have reported that when β-conglycinin subunits (α, α’, and β) and glycinin were heat-treated at pH 7 under a temperature of 100 °C for 30 min, the size and density of the aggregates formed were reduced compared to separate treatment of glycinin. This suggests that β subunits may inhibit the thermal aggregation of glycinin. Under hydrophobic interactions, β-globulin subunits may more easily form soluble complexes with the basic polypeptides of glycinin. Therefore, the ratio of glycinin to β-conglycinin affects the aggregation behavior of soybean protein isolate [67,68]. The ratio of 11S to 7S has also been proposed to reflect the emulsifying ability of soy protein, and this ratio may play a role in emulsion stability [69]. It is believed that the ratio between 11S and 7S depends on the types and the extraction process [70]. 

Despite globulin being the most abundant plant protein, animal proteins with good foaming properties are still used as emulsifiers in many foods [28,70]. The poor functionality of globulin, such as foaming, may be attributed to the formation of aggregates [68]. Some studies have compared the foaming and interfacial properties of albumin from plant proteins (mung bean, yellow pea, and Bambara groundnut) with those of animal proteins (whey protein isolate and egg white protein isolate). The results show that these plant albumins exhibited higher foam stability than their globulins. They are comparable to whey protein-stabilized foam in terms of foam stability and even more stable than egg white protein-stabilized foam [62]. The potential of albumins in foam stability expands the use of plant proteins in creating stable emulsion delivery systems and replacing animal-derived proteins.

### 2.3. Insect Proteins

As a novel protein source, edible insects have a broad market in several Asian and African countries. They possess a high protein content, with Coleoptera species (beetles, grubs) averaging 40.69% protein, surpassing certain plant proteins such as legumes (23.5% protein) [71]. However, in some developed countries, the insect industry primarily focuses on honey and Carmine E120, a red food colorant extracted from female cochineal insects, used in yogurt, confectionery, and beverages. Additionally, Western consumers may be hesitant to directly consume insect proteins for protein supplementation [72]. Therefore, to increase consumer acceptance, separating insect proteins before consumption as a substitute for meat protein products could be an effective approach.

Similar to animal and plant proteins, proteins in insects can stabilize emulsions and form gels. However, research on the functional properties of insect proteins is still in its early stages compared to commonly used protein sources like beans, grains, and milk [73]. Some studies have evaluated the gelling properties of five insect proteins (*Tenebrio molitor*, *Zophobas morio*, *Alphitobius diaperinus*, *Acheta domesticus*, and *Blaptica dubia*) and found that they can form gels of varying strength at pH 7 and pH 10 at a concentration of 30% *w*/*v* [48]. Protein extracted from mealworms has demonstrated stability in oil/water emulsions without apparent droplet coalescence for two months. Mealworm protein with a small amount can produce emulsions with similar microstructures to commercial whey protein-based emulsions [72]. These results highlight the emulsifying properties of mealworm protein, making it a potential substitute or replacement for animal-derived proteins in emulsions. However, further research is needed to understand the structure, conformation, and adsorption of insect proteins at the interface, which plays an important role in their application in food emulsions and gels.

### 2.4. Algae Proteins

Microalgae, primarily used for biofuels, polyunsaturated fatty acids, and pigments, also contain high-value substances such as proteins and polysaccharides, which are often neglected during pigment extraction and the extraction of certain bioactive compounds [74]. When used as a food ingredient, the high protein content of algae (up to 50% w/w) presents a promising alternative protein source [75]. Teuling, Schrama, Gruppen and Wierenga (2019) described the emulsifying properties of soluble protein isolates from algae species *Nannochloropsis gaditana*, *Tetraselmis Impellucida*, and *Arthrospira (Spirulina) maxima*. The algal protein isolates used exhibited similar emulsifying abilities to commercially available whey protein isolates [49].

However, unlike proteins derived from other sources, the presence of non-protein components in algae and cyanobacteria protein isolates, resulting from less-refined protein extraction processes, can affect emulsification performance [76]. Studies have shown that compounds like polysaccharides in algae can interact with proteins and play a significant role in emulsion stability [75]. On the other hand, refined proteins usually enhance the functional properties of the protein, including emulsifying properties [74]. Therefore, when algae proteins are used in food products, these less-refined proteins extracted without organic or other chemical reagents are more readily accepted by consumers. Concurrently, in the process of stabilizing emulsions, the native protein–polysaccharide mixtures in algae can serve as an effective natural substitute for protein–polysaccharide complexes [74].

## 3. Methods for Improving the Emulsifying and Gelling Properties of Proteins

Unlike the liquid-like structure of emulsions, emulsion gels are characterized by a rigid and elastic structure that imparts improved texture and rheological properties. The strong gel network structure of emulsion gels provides enhanced stability for encapsulating bioactive substances within the delivery system [2]. Currently, various approaches are being explored to design and create desirable structures in emulsion gel preparation. These include the following: (i) Protein processing techniques: Several methods such as heat treatment, ultrasound, microwave, high hydrostatic pressure, and cold plasma have been used to improve the emulsifying and gelling properties of proteins. (ii) Composite emulsifiers: Mixing two proteins through electrostatic interactions to create a composite emulsifier. (iii) Polysaccharide-protein systems: The high hydrophilicity of polysaccharides can be used as thickening and stabilizing agents to build mixed polysaccharide-protein-based emulsion gel systems. Table 2 provides an overview of measures to improve protein functionalities in emulsion gels.

### 3.1. Heat Treatment

**Figure 1:**
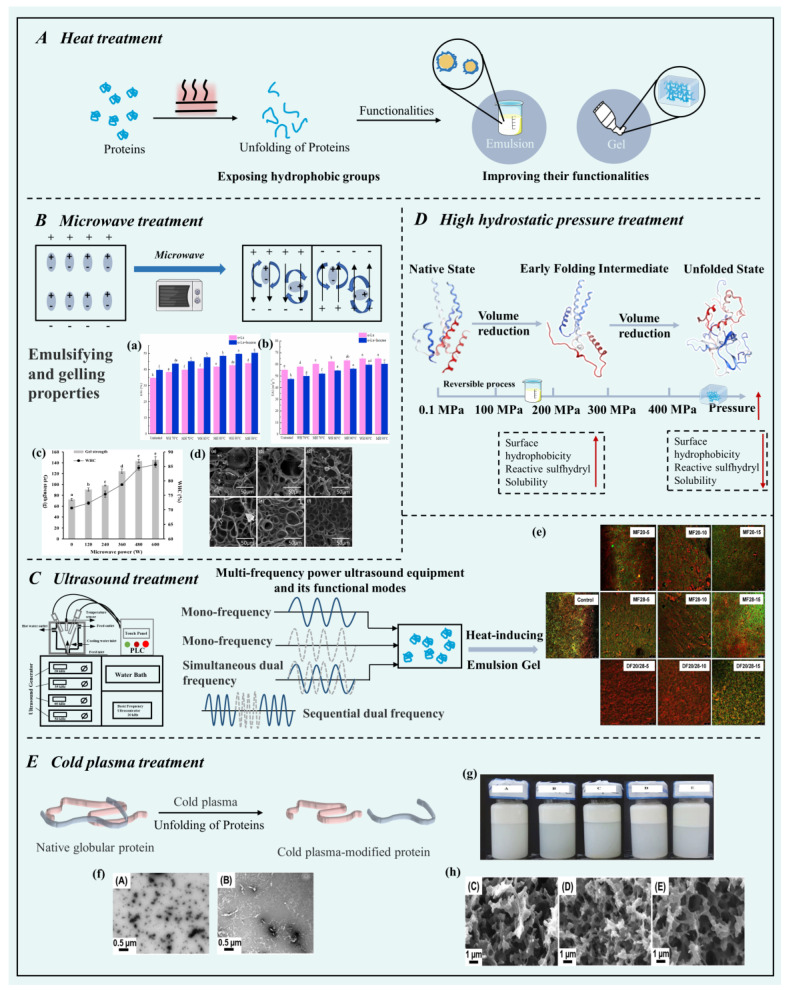
Schematic diagram of physical techniques for improving the emulsifying and gelling properties of proteins: (**A**) Heat Treatment, (**B**) Microwave Treatment, (**C**) Ultrasound Treatment, (**D**) High Hydrostatic Pressure Treatment, (**E**) Cold Plasma Treatment. (Adapted from Refs. [99,100,101,102]). (**a**,**b**) Emulsifying ability (**a**) and emulsion stability (**b**) of α-La and laccase-crosslinked α-La under microwave irradiation heating and water bath heating pretreatments (70, 80, and 90 °C). (Reprinted with permission from Ref. [103]. 2021, Elsevier). (**c**,**d**) Gel strength, water-holding capacity (**c**), and scanning electron microscopy (SEM) images (**d**) of different microwave power pretreatments (0, 120, 240, 360, 480, and 600 W) (from left to right) of laccase-induced soy protein isolate gels. (Reprinted with permission from Ref. [104]. 2020, Wiley). (**e**) Confocal laser scanning micrographs (protein channel in red, magnification 400×) of whey protein-emulsion gel prepared with WPI pretreated with ultrasound at 20 kHz, 28 kHz, and 20/28 kHz (from top to bottom) and time (0, 5, 10, and 15 min) (from left to right). (Reprinted with permission from Ref. [82]. 2019, Elsevier). (**f**) Transmission electron microscopy images of pea protein concentrate suspensions without or with cold plasma treatment (from left to right). (Reprinted with permission from Ref. [61]. 2021, Elsevier). (**g**) Visual images of native and plasma-treated grass pea (*Lathyrus sativus*) protein isolate-stabilized emulsions during storage for 7 d (from left to right: Native; 30 s, 9.4 kVpp; 60 s, 9.4 kVpp; 30 s, 18.6 kVpp; 60 s, 18.6 kVpp). (Reprinted with permission from Ref. [87]. 2020, Elsevier). (**h**) SEM images of pea protein concentrate gels treated by cold plasma at different heating temperatures (70, 80, and 90 °C) (from left to right) for 30 min. CP treatment conditions: 3500 Hz, 10 μs, 10 min, 0–30 kV, and 0–1 A. (Reprinted with permission from Ref. [61]. 2021, Elsevier).

Heat treatment is a widely used physical modification method in food processing to influence protein denaturation and aggregation, thereby promoting the functional properties of proteins and enhancing the functionality of the final product (Figure 1A). The process begins by increasing the thermal mobility of peptide chains, causing the protein structure to unfold and expose hydrophobic groups [95]. Further heat treatment promotes its transition from initial reversible unfolding stacking to irreversible changes accompanied by loss of secondary and tertiary structures of protein molecules, exposing hydrophobic cores and leading to the increased cross-linking of hydrophobic interactions, hydrogen, and disulfide types [96,97]. Proteins with altered structures have been widely used in the food industry due to their improved functionality. For example, thermal pretreatment has been commonly employed to improve protein emulsification and gelling properties in emulsion gels [77]. Tang and Ma (2009) reported a positive correlation between the improvement of emulsifying activity, emulsification stability of kidney bean protein isolate, and increased exposure of protein hydrophobic groups (achieved through heat treatment at 95 °C for 15–30 min) [98]. This increased exposure of the protein hydrophobic groups caused by the preheating treatment enhances the interaction between proteins and oil droplets, a similar result observed in the heat treatment of pea protein [78]. In emulsion-filled protein gels, heat treatment (>65 °C) is traditionally used to induce protein unfolding, causing the exposure of its hydrophobic groups and subsequent aggregation into a three-dimensional network structure with entrapped water by capillary forces. In this process, the hydrogen bonding and ionic bonding involved in the subsequent heating process could further promote aggregation [9,11].

### 3.2. Microwave Treatment

Microwaves are electromagnetic waves with frequencies ranging from 300 MHz to 300 GHz and wavelengths ranging from 1 mm to 1 m [105]. Electromagnetic energy could be converted to heat energy by molecular motion during microwave irradiation (Figure 1B). Due to its high heating efficiency, low energy consumption, and clean operation, microwave treatment has become another widely used heat treatment in the food industry [103]. Compared to traditional heat treatment, microwave treatment has a lesser impact on texture, sensory qualities, and loss of bioactive substances in foods [106]. It has been observed that microwave heating can affect the secondary and tertiary protein structures by breaking non-covalent bonds such as hydrogen bonds and disulfide bonds, thereby facilitating protein unfolding [107,108]. These structural changes in proteins have been widely used to improve their functionality. For example, Zheng, Li, Zhang, Zheng, and Tian (2020) reported that mild microwave treatment (50–100 W) induced the unfolding and stretching of lotus seed protein isolates, exposing more hydrophobic residues, and improving protein adsorption at the oil/water interface [109]. Similarly, microwave pretreatment (300 W/50 s) also improved the emulsifying performance of tartary buckwheat protein after germination [79]. Additionally, higher microwave power is usually required to improve the gel properties of proteins. Increased microwave power may enhance surface hydrophobicity and promote disulfide bond formation, thereby affecting the higher structure of proteins, including tertiary and quaternary structures [110]. Mu et al. (2020) reported that higher microwave power pretreatment led to a higher water-holding capacity, resulting in stronger gel performance [104]. Similar studies have demonstrated that myofibril protein prepared under 500 W microwave exhibited improved springiness and water-holding capacity [110].

### 3.3. Ultrasound Treatment

Ultrasound is widely recognized as an eco-friendly and efficient approach to processing proteins. This section mainly focuses on the effects of mono-frequency ultrasound and dual/multiple-frequency ultrasound on the structure and functional properties of food proteins (Figure 1C). Ultrasound refers to acoustic waves that exceed the threshold of human hearing (>16 kHz) and can be classified into low-frequency (from 16 to 100 kHz, power from 10 to 1000 W/cm2) and high-frequency (100 kHz to 1 MHz, power <1 W/cm^2^) ultrasound [15]. Currently, mono-frequency ultrasound (approximately 20 kHz) is commonly used for physicochemical modification of food proteins [82]. Through the cavitation effect generated by ultrasound, the rapid expansion and rupture of bubbles created by local pressure differences can release high energy, leading to changes in the molecular structure of proteins. Ultrasound treatment can also induce the splitting of water molecules surrounding proteins, generating reactive free radicals (hydrogen and hydroxyl) and non-radical compounds (hydrogen peroxide), which can modify the protein molecular structure through oxidation reactions [111,112]. The alterations in protein structure caused by cavitation of high-energy and reactive free radicals impact the functional properties, especially the emulsifying and gelling properties of the protein.

Previous studies have demonstrated that ultrasound pretreatment improves the emulsifying properties of proteins. Treatment of wheat and soybean isolate proteins with 20 kHz ultrasound resulted in a significant reduction in protein aggregate size and an increase in hydrophobicity. The emulsion prepared from these two proteins exhibited smaller droplet sizes and enhanced long-term stability [113]. Additionally, ultrasound pretreatment also significantly changed the gel properties of whey protein isolate, particularly by increasing gel hardness and strength [114].

Dual- or multi-frequency ultrasound, which utilizes two and/or more kinds of frequencies, is believed to generate stronger cavitation effects compared to mono-frequency ultrasound [115]. Cheng et al. (2019) applied mono-/dual-frequency ultrasound in the pretreatment of whey protein [82]. The dual-frequency ultrasound pretreatment for 10 min showed superior gel performance compared to the mono-frequency pretreatment for 20 min, indicating that dual-frequency pretreatment could achieve better acoustic cavitation capacity while reducing pretreatment time. However, different structural proteins exhibit different responses to different frequencies and modes of operation. A study by Yang et al. (2017) reported that dual-frequency or multi-frequency ultrasound enhanced the structural properties of proteins, and the highest angiotensin-I-converting enzyme inhibitory activity was observed in rice protein hydrolysate under the optimal combination of ultrasound frequencies (20/35/50 kHz sequential triple-frequency ultrasound pretreatment) [116]. Likewise, optimization of ultrasonic parameters for dual-frequency ultrasound has been reported in corn protein through single-factor experiments [117].

### 3.4. High Hydrostatic Pressure Treatment

In recent years, high hydrostatic pressure (HHP) has gained widespread use in the food industry for modifying protein structures. As a non-thermal processing technology, HHP has minimal impact on small molecular substances such as vitamins and certain free amino acids, preserving the nutritional value of food [118]. Previous studies widely believed that the sensitivity of the structure in proteins to HHP is the main change in noncovalent bonds (e.g., hydrophobic and hydrogen bonds), while covalent bonds remain unaffected [119]. Therefore, under the volume compression of HHP, the breakage and recombination of various chemical bonds cause changes in protein structure, showing proteins with altered functional properties (Figure 1D) [120]. For example, HHP treatment has been shown to improve the emulsification and gelling properties of myosin [121], myofibril protein [102], rice bran protein [83], and soy protein isolate [122]. Additionally, factors such as applied pressure, duration of pressure application, and ionic conditions contribute to changes in protein structure under HHP treatment. Many studies have reported that proteins treated at lower pressures (100–200 MPa) exhibit enhanced emulsifying properties. Optimal emulsifying activity and stability have been observed in myosin treated at 150 Mpa [121], while mantle protein-based emulsions processed at 200 Mpa demonstrated similar improvements [120]. When the pressure exceeds 200 Mpa, the hydrophobic forces and ionic bonds responsible for maintaining protein tertiary structure are weakened, thereby inducing protein gelation [121]. Lv et al. (2020) reported the formation of a whey protein isolate gel at 600 Mpa under HHP treatment, and the resulting emulsion gel exhibited a robust gel-like structure and stability [85]. At pressures exceeding 700 Mpa, changes in the secondary structure of proteins can occur [119].

### 3.5. Cold Plasma Treatment

Cold plasma is known as the fourth state of matter (along with solids, liquids, and gases), which is a partially or fully ionized state of a gas. The energy required for ionization can be derived from electricity, electromagnetic waves (such as radio and microwaves), and heat [99]. Cold plasma, in which the electron temperature is high, the temperature with the binding material is close to room temperature. In the biomedical field, this technology is often used for sterilizing heat-sensitive materials [123]. Compared to traditional heating methods, cold plasma has a minimal impact on the sensory and nutritional quality of food. This technology has also been widely used for food surface disinfection, seed germination enhancement, and enzyme inactivation [123,124]. For example, Ahmadnia, Sadeghi, Abbaszadeh, and Marzdashti (2021) reported that the total aerobic mesophilic bacteria and yeast/mold in strawberries treated with cold plasma decreased by 1.46 and 2.75 log CFU/g, respectively [125]. In previous studies, cold plasma increased the germination rate of mung bean seeds by 36.2% [126]. And in fresh-cut apples, the polyphenol oxidase activity was significantly reduced after cold plasma treatment [127]. Additionally, cold plasma is considered a “green” method for structurally modifying proteins and polysaccharides, representing a novel non-thermal processing technology [99,128]. Previous studies have indicated that cold plasma treatment can reduce protein particle size and disrupt protein aggregates, thereby increasing their flexibility for adsorption at the oil/water interface (Figure 1E). For instance, Mehr and Koocheki (2020) reported that grass pea protein isolate treated with higher voltage (18.6 kVpp) and longer duration (60 s) exhibited enhanced surface activity [87]. Emulsions prepared under these conditions demonstrated smaller droplet sizes and increased creaming stability. Sharafodin and Soltanizadeh (2022) demonstrated that soy protein isolate treated with cold plasma at 18 kV for 5 min exhibited the smallest particle size, with improved solubility and emulsifying performance [129]. Furthermore, S. T. Zhang et al. (2021) applied cold plasma treatment to enhance the gelling properties of pea protein. The treated pea proteins formed firm and elastic gels at lower temperatures (80–90 °C) and showed a high water-holding capacity [61].

### 3.6. Interactions between Food-Grade-Biopolymers

#### 3.6.1. Protein–Protein Interactions

Electrostatic assembly based on opposite charges plays an important role in the association of biopolymers. Proteins, being natural biopolymers, possess an amphiphilic nature and structural versatility that allow them to form complexes with polyanions and polycations, such as protein–polysaccharide mixtures [130]. The combination of different protein sources to modify their technical properties has rapidly gained attention [131]. These mixed proteins typically exhibit opposite charges at low ionic strength and within a limited pH range (below their isoelectric point), following the principle of charge compensation [132]. However, electrostatic assembly is not solely driven by electrostatic interactions, as thermodynamics, including entropy gain and negative enthalpy, also contribute as driving forces for complex formation [133]. Currently, composite proteins consisting of basic proteins (positively charged proteins at neutral pH) such as lactoferrin, lysozyme, napin, and gelatin A, and acidic proteins including casein, α-lactalbumin, β-lactoglobulin, ovalbumin, gelatin B, and pea proteins, are being employed to design functional mixed protein products. The combination of highly functional proteins (e.g., casein and whey protein) with plant proteins also holds promise for the development of natural functional foods [33]. To some extent, combining plant proteins with animal proteins could serve as a potential method to partially replace animal proteins in food systems. Zheng et al. (2020) studied the assembly of soy protein isolate and lactoferrin and found that electrostatic interactions and hydrogen bonding were involved in the protein complex [130]. By combining lactoferrin and β-lactoglobulin, these researchers encapsulated vitamin B9 within the protein complex, with optimal encapsulation achieved at approximately 10 mg B9/g protein [89]. X. Y. Zhang et al. (2022) reported that the SPI–WPI composite emulsion gel exhibited improved water-holding capacity and texture, leading to enhanced bioavailability of vitamin E when incorporated in the emulsion gel [33].

#### 3.6.2. Protein–Polysaccharide Interactions

**Figure 2:**
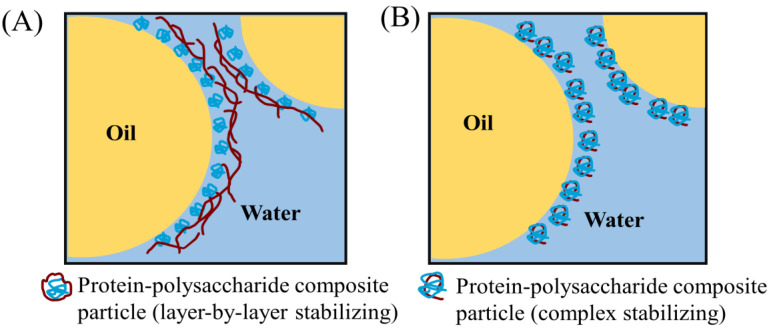
The combination of protein–polysaccharide in emulsion gels: (**A**) Polysaccharide-polysaccharide composite particles (layer-by-layer stabilizing), (**B**) Polysaccharide-polysaccharide composite particles (complex stabilizing) (Adapted with permission from Ref. [138]. 2021, Elsevier).

As natural biopolymers, proteins and polysaccharides have favorable interfacial properties and are abundant in nutrients. Previous studies have widely reported that the stability of emulsions can be improved by combining proteins with suitable polysaccharides [134]. In general, combinations of protein–polysaccharide combinations in emulsion gels are achieved through the following two main approaches (Figure 2): (i) The oil phase is emulsified with the water phase of the pre-mixed protein–polysaccharide composition, followed by the further formation of emulsion gels. (ii) The oil phase is mixed with the water phase containing protein for pre-emulsification, and then polysaccharide is added for secondary emulsification to achieve the ideal emulsion gel (also known as the layer-by-layer technique).

In the first method of pre-mixed protein–polysaccharide emulsification, the interaction between proteins and polysaccharides can occur through electrostatic interaction, hydrophobic interaction, hydrogen bonding, and covalent bonding. Among these forces, electrostatic interaction is the main force for complex formation between charged macromolecules [135]. Natural polysaccharides are mostly negatively charged (except for chitosan). In such cases, positively charged proteins can form protein–polysaccharide complexes with superior functional properties [94]. For example, the electrostatic interaction between lactoferrin (which has a high positive charge on its surface) and xanthan gum (an anionic polysaccharide) has been used to prepare various composite systems and improve emulsification over a wide pH range [93]. However, this electrostatic interaction is still affected by factors such as pH, ionic strength, and the protein/polysaccharide ratio. Some studies have carefully designed these parameters to obtain protein–polysaccharide systems with excellent functional properties. Tavernier et al. (2017) investigated the effects of pH, ionic strength, and the protein/polysaccharide ratio on the ζ-potential, microscopic appearance, and stability, resulting in an optimized protein–polysaccharide composite with high emulsion stability [94]. In some systems where electrostatic complexes are less sensitive to pH or ionic strength variations, non-electrostatic interactions like hydrophobic interactions and hydrogen bonds play a role in the formation and stabilization of protein–polysaccharide complexes [134].

In the layer-by-layer emulsification method, polysaccharides are usually introduced for secondary emulsification in a protein-stabilized emulsion. This process often involves the attraction between polysaccharide molecules and the surface of protein-coated droplets or the thickening effect of polysaccharides [136]. The most commonly employed attractive force is the electrostatic interaction between charged groups on polysaccharide molecules and oppositely charged groups on adsorbed protein molecules. Polysaccharides with opposite charges have been shown to increase the stability of protein-stabilized oil/water emulsions and facilitate the formation of emulsion gels with improved structure. F. G. Liu et al. (2022) used the layer-by-layer emulsification method to add sodium alginate to whey protein isolate-stabilized emulsions, resulting in a more viscous and robust emulsion gel due to the increased cross-linking [92]. Additionally, the presence of polysaccharides significantly influences the structure and stability of the emulsion gel system due to their natural thickening properties. Felix, Romero, and Guerrero (2017) found a strong correlation between the rheological properties of emulsion gels and the concentration of polysaccharides [91]. Soltani and Madadlou (2016) observed that higher pectin content shortened the gelation time of the emulsion and led to a solid-like emulsion [137]. Above all, selecting the appropriate system composition and preparation conditions is beneficial for improving the environmental responsiveness of multilayer interfaces.

## 4. Formation of Protein-Based Emulsion Gels

### 4.1. Heat-Set Gels

Heat-induced gelation is a widely used method in the food industry for inducing gel formation, particularly with globular proteins found in dairy and plant proteins [139,140]. Heat-induced gelation involves heating the emulsion and subsequently cooling it at low temperatures to produce emulsion gels [141]. When the temperature surpasses the denaturing temperature of the globular proteins, they undergo partial expansion, exposing hydrophobic groups within their structure [67]. This exposure facilitates the formation of aggregates through hydrophobic interactions and hydrogen bonds [142]. These aggregates become insoluble aggregates when further heated [140]. The size of these aggregates varies depending on the protein type and external conditions until a three-dimensional network of protein molecules forms throughout the system, ultimately leading to the formation of a gel network [143]. When protein concentrations are sufficiently high, globular proteins can always form gels under heat-treatment conditions in aqueous solutions or dispersions. Whey protein and soy protein isolate, as typical globular proteins, are widely used because of their low price, easy availability, and good stability in oil/water emulsions [144].

The heat-induced gel formation method is often used to prepare bulk emulsion gels, which are often used to prepare fat substitutes based on their properties [145]. The study of heat-induced whey protein isolate-based emulsion gel has excellent applicability in low-fat yogurt because the structure of emulsion gels often brings better sensory quality to yogurt than low-fat substances [6,53]. Based on the interactions between myofibrillar protein and oil droplets, heat-induced myofibrillar protein emulsion gel also has a significant impact on the preparation of sausages, surimi, and other meat substitutes [146]. Similarly, the heat-induced gel also provides a medium method for the development of plant proteins that partially or completely replace animal protein products [147]. However, due to the high-temperature conditions in the preparation process, the thermally induced gel is not suitable for embedding thermally unstable bioactive substances. Nowadays, most tend to use the cold-setting method to prepare emulsion gel to deliver bioactive substances.

### 4.2. Cold-Set Gels

**Figure 3:**
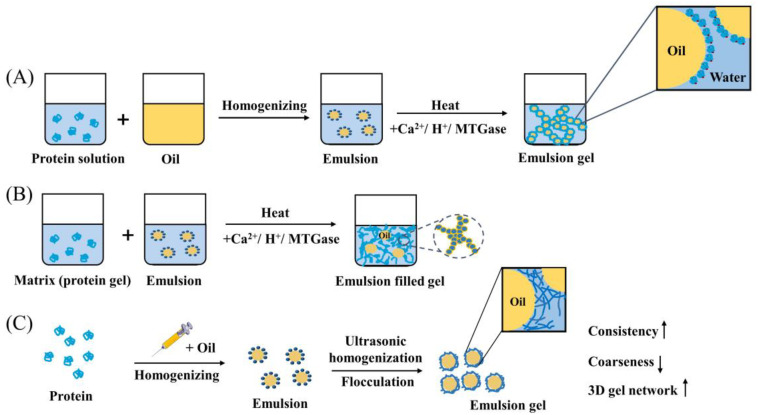
Formation of protein-based emulsion gels: (**A**,**B**) heat/cold-set gels for preparing emulsion gels and emulsion-filled gels, (**C**) ultrasonic homogenization methods in emulsion gels [136,153]. (Adapted with permission from [136,153]. 2023, 2013, Elsevier).

In systems containing globular proteins, the process of cold-gelation of emulsion gels mainly includes the following three processes [77,148]: (i) The protein solution is heated above the thermal denaturation temperature of the proteins to expand their structure. To prevent excessive protein aggregation, certain conditions such as protein concentration, pH, and ionic strength must be controlled. The protein solution should then be cooled to ambient temperature [149,150]. (ii) The preheated protein solutions are emulsified using various homogenization methods such as high-speed shearing, high-pressure homogenization, and/or ultrasonication to prepare O/W emulsions. (iii) Acidifiers (e.g., Glucono-δ-lactone), ions (e.g., Ca^2+^ in the form of CaCl_2_), or enzymes (e.g., transglutaminase) are added to induce the formation of emulsion gels (Figure 3) [18,53]. These gelling agents can improve covalent cross-linking (enzymes) or reduce electrostatic repulsion between proteins [151]. Before gelation, thermosensitive bioactive substances can usually be mixed into the cooled protein emulsions in the second step. Therefore, cold-set gelation is often used for carriers containing thermosensitive bioactive substances [152].

### 4.3. Homogenization Methods

As mentioned earlier, protein-based emulsion gels mainly consist of the following two parts: emulsion preparation and gel formation [18]. Homogenization methods are used to induce gelation; that is, in the process of emulsion gel formation, ultrasonic homogenization and other methods tend to be used to replace the previous heat treatment and crosslinking agent induction [154,155,156]. Homogenization methods are commonly used for emulsion production. Ultrasonic homogenization, for instance, produces strong shear and mechanical forces through cavitation effects, enhancing the emulsifying properties of proteins [157,158,159]. Ultrasonication has also been reported as a pretreatment method to modify the molecular structure of proteins, reducing protein aggregations and increasing hydrophobicity and solubility [160,161,162]. This modification results in a denser gel network structure, improving the gelation properties of proteins [148,163]. Ultrasound is typically used in coordination with other methods, such as homogenization or microfluidization in emulsion systems. However, the higher viscosity of emulsion gels systems can cause chamber clogging in high-pressure homogenizers or microfluidizers [163,164]. Therefore, there is significant potential in using high-intensity ultrasound alone (at frequencies of 100 kHz to 1 MHz and power < 1 W/cm^2^) for the preparation of protein-based emulsion gels [165]. 

## 5. Protein-Based Emulsion Gels as Nutrient Delivery Vehicles

Protein-based delivery systems, such as emulsions, hydrogels, and emulsion gels, are increasingly used for encapsulating, protecting, and delivering bioactive compounds (Table 3). The gel’s unique three-dimensional network structure provides higher stability compared to traditional emulsions [53]. However, traditional hydrogels have limitations in encapsulating lipophilic bioactive substances. Emulsion gels, which contain an oil phase, not only affect the strength and modulus of the gel, but also allow for the dissolution of lipophilic bioactive substances [26]. Therefore, protein-based emulsion gels have become an excellent choice for encapsulating and providing sustained release of lipophilic bioactive ingredients, such as carotenoids (lycopene, curcumin, and astaxanthin), polyunsaturated fatty acids, and oil-soluble vitamins [2].

**Figure 4:**
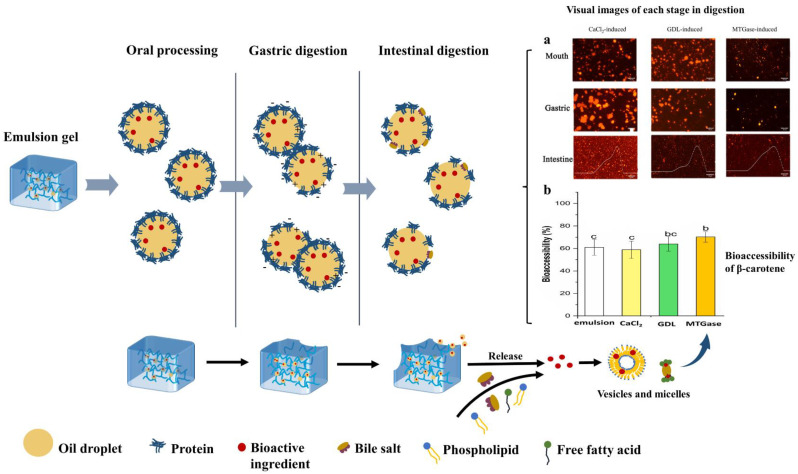
Schematic illustration of the digestion process of emulsion gels stabilized with protein particles, and the release and bioavailability of the encapsulated nutrients: (**a**) Fluorescent microscopy images of β-carotene bulk emulsion gels at the end of each stage in digestion, (**b**) Bioaccessibility of β-carotene emulsions and bulk emulsion gels.(Adapted with permission from [148]. 2022, Elsevier) (Adapted from Refs. [176,177]).

Previous studies have reported that the low absorption and bioavailability of bioactive substances in foods are mainly caused by environmental factors (such as light, heat, and oxygen) and the degradation process of the food matrix in vivo digestion (i.e., oral processing, enzymatic hydrolysis in the stomach and lipolysis by lipase reaction in the intestine) [166]. Therefore, an important factor in characterizing the efficacy of an emulsion gel delivery system is the ability to reach the targeted location of the embedded substances. To predict the bioavailability of the target compound, in vitro simulated gastrointestinal conditions are often used to study the in vivo digestion process. It is currently believed that the absorption process of bio-functional compounds involves degradation of the food matrix, the release of encapsulated bioactives from the gel matrix, interaction with bile salts and endogenous phospholipids, and formation of mixed micelles, ultimately leading to bioavailability (Figure 4) [1,167]. In this process, the compact structure of a gel matrix can significantly slow the diffusion of the digestive enzyme to the surface of the oil droplet, resulting in a slow-release effect in the delivery of bioactive ingredients [168,169]. Guo, Ye, Lad, Dalgleish, and Singh (2016) reported that the structure of gels strongly influences gastric digestion, with a high gel strength slowing down the disintegration of the protein matrix [170]. When the gel structure begins to swell or erode, lipid digestion may be accelerated. To some extent, the structure of emulsion gels has been considered to be adjusted by changing the type and concentration of emulsifiers, oil content, and addition of other components, thus influencing the digestion behavior of the emulsion gel in vivo and controlling the release of bioactive substances [2]. Shao and Tang (2016) reported that increasing the volume fraction of the oil phase in pea protein isolate-stabilized emulsion gels enhanced the gel-like network structure, leading to the sustained release of β-carotene during in vitro simulated digestion [171]. Although some previous studies have reported that insufficient digestion of gel matrices can affect the bioavailability of embedded lipophilic bioactive compounds, the three-dimensional network structure of emulsion gels plays a crucial role in improving the stability of bioactive substances during storage [53]. In a published study, Brito-Oliveira, Bispo, Moraes, Campanella, and Pinho (2017) reported that emulsion gels prepared with soy protein isolate and xanthan gum could improve the stability of curcumin [172]. When the whey protein isolate-stabilized emulsion gels were used as a carrier for β-carotene, the increase in the oil phase could help improve the heat treatment stability and ultraviolet light stability of β-carotene [173]. Therefore, due to the structural designability and high stability of protein-based emulsion gels, they are increasingly used as delivery systems for natural biopolymers in drugs and bioactive ingredients. However, the mechanism underlying their release characteristics and their ability to improve bioavailability still need further study.

**Table 3:** Examples of protein-based emulsion gels for the delivery of various bioactives.

Protein Type	Modification	Ingredient	Homogenization Technology	Gelation Triggers	Bioactive Components	Main Findings	References
Whey protein isolate	High hydrostatic pressure (600 Mpa) to obtain protein gels	Canola oil	Ultra-high-speed homogenization (12,000 rpm for 1 min)		Curcumin	The whey protein isolates stabilized emulsion gels provided a better protection for curcumin (remained about 70% of the initial amount) after storage for 4 h under light. Higher release of curcumin under in vitro intestinal conditions.	[85]
	No	Medium chain triglycerides (MCT)	Homogenization (9,000 rpm for 90 s) and Microfluidization (18000 psi, 2 cycles)	Heat treatment	β-carotene	Emulsion gels systems effectively protected β-carotene. The effect of adding polysaccharide on bioactive substances was related to the effect of polysaccharide on gel structure.	[54]
	Heated treatment (90 °C for 5 min)	Sunflower oil	Homogenization (10,000 rpm for 1 min) and Microfluidization (50 MPa, 3 passes)	Acidification treatment (Glucono-δ-lactone)	Modulate volatile release (propanol, diacetyl, pentanone, hexanal, and heptanone)	Emulsion-filled protein gels slowed the volatile release by varying the rheological properties of the gels.	[11]
β-lactoglobulin	Heated treatment (85 °C for 45 min)	Sunflower oil	Homogenization (20,000 rpm for 2 min) and Microfluidization (100 MPa for the first-stage, 10 MPa for second-stage)	Addition of ions (Ca^2+^)	α-tocopherol	The gel structure of cold-set β-lactoglobulin emulsion gels improved the chemical stability of α-tocopherol.	[9]
Egg yolk granule protein	No	Sunflower oil	Homogenization (15,000 rpm for 1 min)	Addition of ions (Ca^2+^)	β-carotene	A more uniform and dense emulsion gel structure of pH 4.0 than pH 7.0 improved storage stability, FFA releasing, and chemical stability of β-carotenes.	[174]
Soybean protein isolate	Soybean protein isolate (final concentration 7%) with pectin (final concentration 3%)	Soybean oil	Homogenization (20,000 rpm for 5 min)	Ultrasonication (0, 150, 300, 450, and 600 W, for 15 min) and then heat treatment	β-carotene	High intensity ultrasound treatment improved the stability of emulsion gels. High intensity ultrasound treatment enhanced the stability of β-carotene digestion in vitro digestion.	[8]
	Soybean protein isolate (6.0%, w/w) with sugar beet pectin (2.0%, w/w) and then heated treatment (85 °C for 15 min)	Medium-chain triglycerides	Homogenization (10,000 rpm for 3 min) and Microfluidization (30 MPa, 5 passes)	Acidification treatment (Glucono-δ-lactone) and then added laccase/Enzyme treatment (Transglutaminase) and then added laccase	The hydrophilic phase was loaded with the riboflavin, and the lipophilic phase (MCT) was loaded with β-carotene	Compared with the emulsion gel induced by glucono-δ-lactone, the structure of the emulsion gel induced by transglutaminase was denser. The release of both β-carotene and riboflavin was regulated by the gel network induced by different induction methods.	[26]
	Heated treatment (90 °C for 30 min)	Sunflower oil	Homogenization (14,000 rpm for 3 min) and Ultrasonication (20 kHz, 90 W for 3 min)	Addition of ions (Ca^2+^)/Acidification treatment (Glucono-δ-lactone)/Enzyme treatment (Transglutaminase)	β-carotene	Bioaccessibility of β-carotene in bulk emulsion gels was higher than that of in emulsions. The addition of different coagulants affected β-carotene emulsion gels.	[148]
Zein	Zein with heated (150 °C) food-grad glycerol solutions	Soybean oil	Soybean oil was preheated to 95 °C and was added to the heated zein-glycerol mixed solutions and homogenization (10,000 rpm for 3 min)		β-carotene	The formation of emulsion gels significantly enhanced the UV photo-stability of β-carotene. The addition of β-carotene delayed the oxidation of the corresponding oil phase during storage.	[175]

## 6. Conclusions

The designability and high stability of protein-based emulsion gels make them an attractive option for protecting bioactive substances. This study systematically discussed the formation and modification of protein-based emulsion gels. To better release and bioavailability of the encapsulated nutrients, we believe that the designable structure of emulsifiers in these emulsion gels plays a crucial role in the delivery system. This includes improving the proteins’ emulsifying and gelling properties, constructing dual-protein-based emulsion gels, and adding polysaccharides as thickening and stabilizing agents. These protein-based emulsion gels with high nutritional value, excellent functional properties, high biocompatibility and biodegradability can be better used to prepare delivery materials in food and medicine. Additionally, due to the complex nature of protein-based emulsion gel systems during digestion, it is essential to further explore the relationship between the structural changes of the gel and the release mechanisms of bioactive substances. This exploration will contribute to enhancing the bioavailability of the bioactive substances by improving their stability.

## Figures and Tables

**Table 1:** Summary of protein components and their functionalities from various sources.

Protein Sources	Proteins	Protein Structure	Protein Fractions	Isoelectric Point	Molecular Weight (kDa)	Functional Properties	References
Dairy products	Casein	Unstructured, flexible random coil protein	α_s1_-casein	4.4–4.8	23.6	Excellent interfacial stability, emulsifying, gelling, foaming, and water-binding properties	[28,36]
			α_s2_-casein	4.9	25.2		
			β-casein	4.8–5.0	24.0		
			κ-casein	~3.5	19.1		
	Whey protein isolate	Globular protein	β-lactoglobulin	5.35–5.49	18.3	Excellent emulsifying, gelling, foaming, and water-binding properties	[37,38]
			α-lactalbumin	4.2–4.5	14		
			Serum albumin	5.5	65		
			Lactoferrin	9.5–10.0	76.5		
Legumes	Soybean protein	Complex globular protein	β-conglycinin (7S)	4.6	150–200	The foaming and emulsifying properties of untreated soy protein isolate are not as good as those of animal proteins	[27,39]
			Glycine globular protein (11S)	4.6	300–380		
	Pea protein	Complex globular protein	Legumin 11S	4.8	330–410	Having functional properties similar to soy protein, such as emulsifying properties	[40,41]
			Vicilin/convicilin 7S	5.5	150/180–210		
Cereals	Rice protein	Complex globular protein	Albumin	4.1	10–100	Low foaming capacity but high foaming stability. Emulsifying performance is similar to foaming properties	[42]
			Globulin	4.3/7.9	16–130		
			Glutelin	4.8	51–57		
			Prolamin	6.0–6.5	10–16		
	Wheat Protein	Complex globular protein	Glutenin	7.5	30–140	Poor emulsifying properties when compared to other vegetable proteins (such as zein)	[43]
			α-gliadin/β-gliadin/ γ-gliadin/ω-gliadin		25–35/30–35/35–40/55–70		
Seeds	Flaxseed	Complex globular protein	12S linen	4.75	252–298	High emulsifying activity and foaming capacity	[44]
			2S conlinin		16–18		
	Sunflower	Complex globular protein	11S globulin (helianthinin)	4.5	300–350	Poor gel-forming properties and similar emulsifying properties to corresponding soy-based emulsions	[45]
			2S albumin		10–18		
Insects	*Patanga succincta* and *Chondracris roseapbrunner*	ND	ND	4.0	20–250	Poor emulsifying and foaming properties, but foaming stability was better than bovine serum albumin.	[46]
	*Tenebrio molitor*	ND	ND	ND	14–100	Good gel-forming capacity	[47,48]
Algae	*Arthrospira maxima*	ND	ND	ND	36–112	Good interfacial stability, and emulsifying properties	[49]

ND: not detected.

**Table 2:** Methods for improving the emulsifying and gelling properties of proteins in emulsion gels.

Modification Approach	Treatment Condition	Protein Type	Modified Characteristics	References
Physical techniques	Heat treatment	Soybean protein isolate	Emulsifying properties↑ The strength of the emulsion gels↑ (when induced by glucono-δ-lactone and CaCl_2_)	[77]
		Pea protein isolate	Emulsion-forming ability↑ (pH = 7.0)	[78]
	Microwave treatment	Germinated tartary buckwheat protein	Solubility and Emulsifying properties↑	[79]
		Soybean protein isolate	Texture, water-holding, and hydration properties of the emulsion gel↑	[80]
	Ultrasound treatment	Soybean protein isolate	Solubility and oil binding capacity↑ Rheological properties of emulsion gels↑	[81]
		Whey protein	Mechanical properties of emulsion gels↑	[82]
		Soybean protein isolate	Particle size↓ and the water holding capacity↑ of the emulsion gels Chemical stability and bioaccessibility of β-carotene in the emulsion gels↑	[8]
	High hydrostatic pressure treatment	Rice bran protein	Solubility, emulsifying properties, and foaming properties↑	[83]
		Myofibrillar protein	Solubility↑ Microstructure and hardness of myofibrillar protein gel↑	[84]
		Whey protein isolate	The gel structures and creaming stability of the Pickering emulsion gels↑	[85]
	Cold plasma treatment	Peanut protein	Protein solubility↑ Emulsion stability↑ Water holding capacity of the protein gel↑	[86]
		Grass pea protein isolate	The interfacial and emulsifying properties↑	[87]
Protein-protein interactions	Electrostatic interactions	Soybean protein isolate–whey protein isolate composite	Water-holding capacity and texture of the composite protein-based emulsion gel↑ The bioavailability of vitamin E↑	[33,88]
		Lactoferrin and β-lactoglobulin composite	The encapsulate of B9 by the Lactoferrin–β-lactoglobulin coacervates↑	[89]
Protein-polysaccharide interactions	Thickening with polysaccharides	Soybean protein isolate–flax gum	Rheological properties, thermal properties, microstructure, and gel properties of the SPI-FG based emulsion gels↑	[90]
		Crayfish protein–xanthan gum	Emulsion stability↑	[91]
		Whey protein isolate–sodium alginate	The gel strength and viscosity of the double-crosslinked emulsion gels↑	[92]
	Electrostatic interactions	Lactoferrin-xanthan complex	The stabilization of Lactoferrin-xanthan complex by adding xanthan↑	[93]
		Soybean protein isolate–κ-carrageenan complex	The complexes-based emulsion and oleogel stabilization↑	[94]

## Data Availability

Not applicable.
